# C_1_ inhibitor in canine intravascular hemolysis (C_1_INCH): study protocol for a randomized controlled trial

**DOI:** 10.1186/s12917-019-2220-2

**Published:** 2019-12-30

**Authors:** Robert Goggs, Erica Behling-Kelly

**Affiliations:** 1 Department of Clinical Sciences, Cornell University College of Veterinary Medicine, Ithaca, NY 14853 USA; 2 Department of Population Medicine and Diagnostic Sciences, Cornell University College of Veterinary Medicine, Ithaca, NY 14853 USA

**Keywords:** Dogs, IMHA, C1-INH, Ruconest, Complement, Intravascular, Hemolysis, Randomized clinical trial

## Abstract

**Background:**

Immune-mediated hemolytic anemia (IMHA) is a common disease that affects all breeds of dogs and is associated with significant morbidity and mortality. Intravascular hemolysis of erythrocytes in IMHA is caused by complement activation and is often fatal. No current treatments target complement activation in canine IMHA. Human C_1_ esterase (C_1_-INH) reduces canine complement-mediated hemolysis in vitro, and a recent pharmacokinetic analysis of an FDA licensed formulation of C_1_-INH in dogs confirmed that a 50 IU/kg dose of C_1_-INH is safe to administer to dogs, and effectively inhibits canine complement mediated hemolysis ex-vivo. The C_1_INCH randomized controlled trial will evaluate the efficacy of this drug in dogs with intravascular hemolysis.

**Methods:**

We will conduct a multicenter, placebo-controlled double-blind randomized clinical trial of C_1_-INH in dogs with intravascular hemolysis due to IMHA. We will randomize 18 dogs to receive three doses of intravenous C_1_-INH or saline in 24 h. Immunosuppressive and antithrombotic therapies will be standardized. Primary outcome measures will be changes in plasma free hemoglobin, serum concentrations of LDH, bilirubin, and haptoglobin. Using patient samples, we will evaluate complement activation in canine IMHA using a novel C5b-9 ELISA assay, flow cytometric detection of C3b on RBC, and by measurement of residual plasma complement activity. Secondary outcome measures will be survival to hospital discharge, duration of hospitalization, number and volume of red blood cell transfusions, and rescue therapy requirements. We will monitor dogs for adverse drug reactions. Sample size was estimated from pilot data on LDH and hemolysis index (HI) in dogs with IMHA. To detect 2-way differences between the upper and lower 50% of the LDH and HI values of equivalent size with 80% power at *P* < 0.05 will require 9 dogs in each arm.

**Discussion:**

We anticipate that IV administration of C1-INH will significantly inhibit complement mediated hemolysis in dogs with intravascular IMHA, as determined by blood biomarker measurements (decreased plasma hemoglobin, LDH and bilirubin, increased haptoglobin). We expect this will translate into significant reductions in transfusion requirements and duration of hospitalization.

**Trial registration:**

This trial has been prospectively registered with the AVMA registry (AAHSD005025).

## Background

Immune-mediated hemolytic anemia (IMHA) is among the most common immunologic disorders in dogs [1]. The cause of primary IMHA is unknown, but this autoimmune condition is characterized by the production of autoantibodies directed at endogenous antigens expressed on the surface of erythrocytes (RBC) [2]. In the most severe form of this disease, RBC are destroyed while still in the bloodstream, a process termed intravascular hemolysis. Intravascular destruction of RBC is profoundly inflammatory, prothrombotic, and leads to a severe reduction in the blood oxygen carrying capacity. This can ultimately lead to thromboembolic disease, shock and organ dysfunction that contribute to both morbidity and mortality [3–5]. Despite years of research and advances in the diagnosis of IMHA, treatment options have stagnated. The mainstays of therapy remain non-specific immunosuppressive drugs. There is a crucial need for novel therapies to improve outcomes in affected dogs. Treating animals with IMHA is challenging and the disease may be associated with a mortality rate of up to 70% [6–12]. Most dogs that succumb die within 2 weeks of presentation, suggesting that a narrow window exists to exert control [9, 10].

Intravascular hemolysis is mediated by the complement system, a series of enzymatic plasma proteins activated by interactions with antibody-coated cells. The end result of complement activation is the formation of the membrane attack complex (MAC), also known as C5b-9. The C5b-9 complex binds to cell membranes near sites of antibody deposition and perforates the cell membrane, resulting in lysis. In a related human disease, termed paroxysmal nocturnal hemoglobinuria (PNH) uncontrolled activation of the complement system causes bouts of intravascular hemolysis [13, 14]. Recently, treatment of PNH has been revolutionized by introduction of pharmaceutical complement inhibitors [15]. Although the underlying cause for PNH and intravascular IMHA is different, the final pathway of these diseases involving complement-mediated hemolysis is the same. Both humans with PNH and dogs with IMHA are at significantly increased risk of thromboembolic disease [16, 17]. In addition to directly causing hemolysis, complement activation also activates the intrinsic coagulation pathway. This may contribute to the increased thrombotic risk associated with IMHA and offers another means by which complement inhibition may reduce disease severity [17]. Given these similarities, we hypothesize that complement inhibition will effectively treat canine IMHA, and in particular those dogs with intravascular hemolysis.

We have previously identified that an FDA approved recombinant human C1 esterase inhibitor (C1-INH) preparation (Ruconest, Pharming Healthcare Inc., Bridgewater, NJ) inhibits canine complement. In a recent pharmacokinetic study, we confirmed that it can be safely administered to dogs and have demonstrated that a 50 IU/kg dose effectively inhibits complement mediated hemolysis ex vivo. The potential utility of C1-INH is further supported by evidence that C1-INH is active against the main activator of complement (C1) in both the fluid and solid phase (when the C1 has already bound to the cell membranes) meaning that the drug should work when the disease is clinically evident [18]. Data from transplantation studies in dogs suggest that a 50 IU/kg dose of C1-INH protects against complement-mediated ischemia-reperfusion injury in an allogenic lung transplant model [19], and reduces endotoxin-induced pulmonary dysfunction and coagulation activation [20]. In those studies, C1-INH administration ablated complement-mediated effects for at least 3 h. C1-INH has also been used successfully in people to manage autoimmune hemolytic anemia [21, 22]. These data suggest that C1-INH could transform how we manage canine IMHA patients. The clinical effects of complement inhibition are likely to be immediate, which we envisage will enable the stabilization of severely affected dogs, reducing transfusion requirements and increasing survival.

## Methods/design

### Hypothesis

We hypothesize that intravenous C1-INH reduces intravascular hemolysis in dogs with IMHA.

### Study objectives

1. To assess efficacy of C1-INH for management of intravascular IMHA in dogs
2. To assess the frequency of adverse drug reactions to intravenous C1-INH

### Design

Multicenter, randomized, double-blind, placebo-controlled, interventional trial.

### Study settings

Academic veterinary teaching hospital (NY, USA) and private multispecialty referral practice (CT, USA).

### Eligibility criteria

- Age between 0.5y-10y
- Any sex or neuter status
- Any breed
- Between 4 and 28 kg

### Inclusion criteria

Primary intravascular IMHA based on anemia (< 37%) AND one or more of:

- Positive in-saline agglutination test
- Positive Coombs’ test (if not auto-agglutinating)
- Moderate to marked spherocytosis established by a clinical pathologist

and

• Evidence of intravascular hemolysis (hemoglobinemia or hemoglobinuria).

### Exclusion criteria

- Platelet count < 30,000 cells / μL
- Underlying associated disease process e.g. Infectious disease, sepsis, neoplasia, etc.
- Prior corticosteroids for ≥72 h or other prior immunosuppressive therapies e.g. mycophenolate mofetil

### Standardized diagnostic evaluation

- Complete blood count (reviewed by a clinical pathologist), serum chemistry panel, urinalysis
- In-saline agglutination test, Coombs’ test (unless auto-agglutinating)
- Three-view thoracic radiographs, abdominal ultrasound
- Tick-borne disease serology or PCR testing

### Baseline characteristics and stratification

To enable comparison of the two groups at baseline and allow for illness severity scoring and stratification, we will measure baseline characteristics of dogs recruited to the study at the time of randomization. These characteristics will include patient age, sex, neutering status, breed, bodyweight, clinicopathologic data and a calculated IMHA-specific illness-severity score [12].

### Study intervention

- Ruconest® 50 IU/kg slow IV push (over 5 min) every 8 h for 24 h

or

- Equal volume 0.9% saline placebo slow IV push (over 5 min) every 8 h for 24 h

A saline placebo was selected to account for the placebo effect potentially associated with administration of a “study drug” infusion i.e. to account for any effects from this treatment and from enrollment of the patient in a clinical trial that do not depend on administration of C1-INH.

### Standardized therapy

The following therapies will be provided to every patient from randomization until hospital discharge:

- Dexamethasone sodium phosphate (solution for injection) 0.25 mg/kg IV q24h (Max. dose 8 mg)

or

- Prednisolone (oral tablet) 2 mg/kg PO q24h (Max. dose 60 mg)

and

- Mycophenolate mofetil 10-15 mg/kg PO q12h (liquid suspension or to closest capsule size)
- Clopidogrel (oral tablet) 2 mg/kg by mouth q24h

### Patient sampling

Serial patient sampling will be performed per Table 1. Blood samples for complete blood counts and serum chemistry panels will be collected daily and analyzed by the Cornell Clinical Pathology Service. Blood samples will be taken at admission (day 1), and then daily until discharge or until day 5. At each time point blood will be collected into plain, EDTA, citrate and heparin vacuum tubes (total 8.1 mL). For a 4 kg dog, this represents < 2.5% blood volume, an acceptable single blood draw for human pediatrics. Vital parameters and clinical signs will be recorded daily to enable illness severity scoring and adverse drug reaction identification.

**Table 1:** A schematic of the study timeline including the standard protocol items as defined by the 2013 SPIRIT statement [23, 24]

		Screening	Study period						Follow-up period		
		T0	Day 1	Day 2	Day 3	Day 4	Day 5	Discharge	Day 7	Day 14	Day 28
Enrollment	Eligibility screen	x									
	Informed consent	x									
	Randomization and allocation		x								
Intervention	Standardized workup		x	x	x						
	Standardized therapy		x	x	x	x	x		x	x	x
	Drug or placebo (q8h)		x, x, x								
Assessment	Complete blood count		x	x	x	x	x				
	Serum chemistry (LDH, HI, Hb, bilirubin)		x	x	x	x	x				
	C5b-9 complex		x	x	x	x	x				
	Residual plasma complement		x	x	x	x	x				
	Serum haptoglobin concentration		x	x	x	x	x				
	Erythrocyte bound C3b		x	x	x	x	x				
	Duration of hospitalization							x			
	Red cell transfusion requirements							x			
	Rescue therapy requirements							x			
	Survival (Alive, died, euthanized)							x	x	x	x
Follow-up	Telephone follow-up								x	x	x

### Primary outcome measures

All primary outcome measures will be assessed from admission (day 1) until discharge or day 5

- Decrease in serum activities of LDH and in the hemolysis index (HI)
- Decrease in serum concentrations of hemoglobin, bilirubin, and C5b-9 complex
- Increase in serum haptoglobin concentration
- Decrease in erythrocyte bound C3b and residual plasma complement activity

Statistically significant decreases in LDH, HI, hemoglobin or bilirubin over time in the treatment group that are not present in the placebo group will be considered positive outcomes for this trial. The study is powered to detect a difference of 170 HU in the hemolysis index and 263 U/L difference in LDH activity.

Serum LDH activity, HI and bilirubin concentration will be measured with a laboratory chemistry analyzer (Cobas, Roche, Indianapolis, IN). Plasma hemoglobin and serum haptoglobin concentrations will be measured by spectrophotometry. For serum haptoglobin determination, a reduction step with sodium hydrosulphite will be performed and spectrophotometer reads at multiple wavelengths will be used to quantify free hemoglobin, free haptoglobin, and complexes of hemoglobin:haptoglobin [25]. These will be verified by gel electrophoresis and staining with the heme-specific stain, ortho-tolidine. Serum C5b-9 complex concentrations will be measured using a commercial ELISA (MicroVue SC5b-9 EIA Kit, Quidel, San Diego, CA). Erythrocyte bound C3b will be measured by flow cytometry using a FITC-conjugated antibody (Anti-C3 polyclonal antibody, Creative Diagnostics, Shirley, NY) [26]. Residual plasma complement activity will be measured by a hemolysis inhibition assay using antibody-coated sheep erythrocytes as previously [27]. Any residual samples will be stored for a period of at least 3 years for additional future analyses and may be made available to the Cornell Biobank (https://www.vet.cornell.edu/departments/centers/cornell-veterinary-biobank).

### Secondary outcome measures

#### Survival to hospital discharge

Mortality due to IMHA will be defined as any death that is ascribed to the pathophysiologic consequences of IMHA including in-hospital or out-of-hospital cardiopulmonary arrest, severe intractable anemia, failure to respond to therapy, thromboembolic disease. Euthanasia will be treated as non-survival for the purposes of outcome assessment.

#### Duration of hospitalization

Determined as the difference between the date of randomization and the date of hospital discharge. Where clinicians would have discharged patients earlier than was feasible for clients to have collected them they will record the date the patient was capable of being discharged.

#### Number and volume of red blood cell transfusions

Number of red blood cell units will be defined as the number of packed red blood cell units (whole or half) that a patient receives during their initial period of hospitalization. We will also record the date and time of administration. Volume (mL/kg) of red blood cell transfusions will be calculated as the sum of the volume of packed red blood cell units administered divided by the patient bodyweight at the time of randomization.

#### Adverse drug reactions

Any and all adverse drug reactions in each of the groups will be recorded. In particular, we will monitor for signs of the most common complications document in humans including anaphylaxis (increased heart rate, respiratory rate, rectal temperature, urticaria, facial swelling, stertor, stridor, breathing difficulties) and for gastrointestinal signs such as vomiting and diarrhea.

#### Rescue therapy

Defined as open-label administration of non-standardized additional therapies. The number and type of rescue therapies will be recorded. Rescue therapy will be permitted if:

- A patient has been hospitalized for > 5 days without evidence of a response to therapy

(no improvement in hematocrit, or ongoing agglutination, or Coombs’ positivity, or spherocytosis)

- A patient requires more than 4 packed red blood cell transfusions within a 5-day period
- A patient develops thrombotic complications necessitating additional antithrombotic drugs
- Rescue therapy may be considered if other events not covered above occur that the attending clinician determines necessitates additional therapeutic intervention.

### Data collection

Sample data collection forms are available as (Additional files 1 and 2). Records will be collected in paper format, manually curated for accuracy and completeness and stored in an electronic database (Excel for Mac, Microsoft, Redmond, WA).

### Randomization, stratification, allocation concealment

Permuted block randomization (4 dogs per block) will be performed separately at each center. Stratification will be performed using previously published illness-severity scoring [12]. Randomization will be conducted by research technicians at the two study sites using a randomization service to assign patients to each group (Sealed Envelope Ltd., 2018. https://www.sealedenvelope.com/simple-randomiser/v1/). Clinicians, clients and investigators will be blinded to treatment allocation. Study drugs will be distributed to both sites from the licensed pharmacy at Cornell (Ithaca) in containers labelled with unique codes, linked to the blinded treatment allocation coding. Research technicians at the two study sites will responsible for blinding and unblinding. Unblinding will be permitted if adverse drug reactions necessitating drug discontinuation should occur.

### Recruitment

Patients will be recruited for a 36-month period. The trial will seek to enlist additional centers if case recruitment rates are < 1 case per month at either center for 3 consecutive months. The study will provide funds to support the standardized diagnostic evaluations and all of the study blood work will be performed at zero cost to clients. It is expected that this financial support may encourage recruitment to the study. The study period is short and most of the data will be gathered during hospitalization. As such, study retention is not expected to be a limiting factor.

### Sample size justification

There are no equivalent studies in human medicine on which to base sample size calculations. Sample size was therefore estimated from pilot data on LDH and hemolysis index in dogs with IMHA, divided into upper and lower 50% bands, using an online calculator (SISA-Sample Size, Quantitative Skills http://www.quantitativeskills.com/sisa/calculations/samsize.htm). The mean ± SD HI values were 7.6 ± 5 (HU) and 177.5 ± 234.7 (HU), and the mean ± SD LDH values were 83.6 ± 32.4 (U/L) and 346.3 ± 187 (U/L) respectively. To detect 2-way differences of equivalent size with 80% power at *P* < 0.05 would require 9 dogs in each arm. We will therefore recruit 18 dogs in total (9 dogs in each arm).

### Data analysis plan

Prior to test selection, data will be assessed for normality and descriptive statistics calculated as appropriate. Baseline characteristics will be compared using Student’s t-test or the Mann-Whitney U test. Primary outcome measures (analyte concentrations) will be analyzed as dependent variables using mixed-effects linear models that account for patient number, treatment arm and day of hospitalization. Day of hospitalization and treatment arm will be treated as fixed effects, with patient number treated as a random effect. These models will be used to control for repeated measurements on patients over time. Log transformations will be applied if necessary to ensure model assumptions of normality and homogeneity of variance are satisfied. Pairwise comparisons of analyte concentrations over time and between treatment arms will be performed with Bonferroni’s correction for multiple comparisons. Hospitalization duration and red cell transfusion requirements will be compared between treatment arms with Student’s t-test or the Mann-Whitney U test. Binary variables such as survival, rescue therapy and drug reactions will be analyzed using 2 × 2 contingency tables and Fisher’s exact test. Analyses will be performed on an intention-to-treat basis. No interim analysis is planned. Statistical analysis will be conducted using commercial software (Prism 8.3, GraphPad Software, La Jolla, CA; SPSS 26.0, IBM Corp, Armonk, NY), with alpha set at 0.05. Due to the small size of this study, a data monitoring committee is not necessary. Similarly, no interim analyses are planned.

### Anticipated results

We anticipate that intravenous administration of C1-INH will significantly inhibit complement mediated hemolysis in dogs with intravascular IMHA, as determined by blood biomarker measurements (decreased plasma hemoglobin, decreased LDH, decreased bilirubin, increased haptoglobin). We expect that this will translate into significant reductions in transfusion requirements and duration of hospitalization. We anticipate that intravenous C1-INH will be safe to administer. We expect that the C5b-9 ELISA will enable detection of complement activation in dogs with IMHA.

### Ancillary and post-trial care

There are no general provisions or plans for therapy or compensation for any harm related to trial participation. The risks and benefits of study participation and the financial expectations will be explained to pet owners during the consenting process. Specific harms relating to trial participation will be assessed and addressed locally on a case-by-case basis.

### Drug availability

C1-INH is available as the FDA approved formulation Ruconest®. This drug is available from wholesalers including ASD Healthcare (part of AmerisourceBergen) and from Cardinal Health.

### Study timeline

November 1st 2019 – October 31st 2020: Recruitment year 1 – Enrollment of dogs 1–8 anticipated.

November 1st 2020 – October 31st 2021: Recruitment year 2 – Enrollment of dogs 9–16 anticipated.

November 1st 2021 – December 31st 2021: Recruitment year 3 – Enrollment of dogs 17–18 anticipated.

January 1st 2022 – March 31st 2022: Laboratory assays, data analysis.

April 1st 2022 – October 31st 2022: Manuscript preparation and abstract presentation.

### Publication plans

We will present our results at an international scientific meeting, submit the study for publication in a peer-reviewed veterinary journal and in an article for the lay press (e.g. *Cornell Dog Watch* or *The Bark*). Significant protocol deviations will be published alongside study results. Study investigators that recruit and manage cases at each site will be eligible for study authorship. These investigators will be expected to contribute to data analysis and assessment and to manuscript authorship or editing per journal policies.

## Discussion

This multicenter interventional trial will investigate a novel complement inhibitor for the management of canine IMHA. Various challenges have been encountered during the design, organization and initiation of the study, and it is possible that additional unforeseen obstacles to implementation may arise during the trial itself. Pre-existing and preliminary data that supported the grant application for this study suggest that C1-INH is a safe and effective complement inhibitor in dogs. While there is data to suggest that C1-INH is effective in suppressing human immune-mediated hemolytic anemia, the drug has not been previously tested in dogs with the disease. It is hoped that the drug is an effective therapy for the disease and that the study protocol will enable important treatment effects to be identified. It is possible that alterations to the study protocol may be necessary as the trial proceeds, for instance to ensure patient safety or to guard against futility. Publication of the study protocol is an important step towards maximizing transparency and the trial investigators are committed to highlighting and explaining any deviations from these plans in future study publications.

The study will be performed in a double blind manner which poses certain practical issues because the study drug is lyophilized, while the placebo is liquid. To circumvent this, study investigators will obtain the drug from the hospital pharmacy in a ready to use form i.e. reconstituted if the patient is receiving study drug. It is anticipated that some of the patients enrolled in this study will be recruited outside of normal pharmacy hours. To address this, labeled study drug or placebo packages will be available within an automated medication dispensing system that can be accessed at any time. Licensed veterinary technicians will be able to access these study packages and will ensure that the attending clinicians and study investigators remain blinded. The study website, study email account and randomization list were created by a pharmacist at the primary study center to ensure that the principal study investigators were not aware of potential patient allocations.

One potential issue that a multiyear study such as this can encounter are changes in practice, therapeutic options or disease prevalence. These factors could make the study difficult to complete, or potentially reduce the utility of the findings if other effective treatment options become available in the interim. Conducting the study in a timely manner will be essential to maximize the potential benefit of the results. Likewise, a multiyear study must also account for changes in costs due to inflation and careful budgeting is necessary to minimize cost-overruns.

Conducting a multicenter interventional study poses potential operational challenges. In particular, the costs involved in administering the study, analyzing patient samples or performing diagnostic evaluation may be dissimilar between centers. Potential options to address these issues include specific, unified and pre-determined study pricing or research discounting or the use of a client cost rebate mechanism to help defray any costs incurred due to study enrollment. Extensive discussions and negotiations in advance of study initiation may be required to establish the necessary study agreements. Working with committed, and enthusiastic colleagues is essential for successful collaborations such as this.

## Supplementary information

**Additional file 1: **C1INCH trial checklist (BMC Vet Res). A checklist for the C1INCH trial listing inclusion and exclusion criteria, sample requirements and the necessary steps for patient enrollment and randomization.

**Additional file 2: **C1INCH trial forms. Data collection sheet for the C1INCH trial including instruments for collection of follow-up metrics.

## Data Availability

Upon publication of the study, the datasets generated and analyzed will be available from the corresponding author on reasonable request for academic (non-profit) purposes. Biologic specimens collected as part of this trial will be stored at the study institution for a period of 3 years. Upon collection, these specimens will be the property of Cornell University.

## Abbreviations

AAHSD: AVMA Animal Health Studies Database
AVMA: American Veterinary Medical Association
C1-INH: C1 esterase inhibitor
HI: Hemolysis index
HU: Hemolysis units
IMHA: Immune-mediated hemolytic anemia
LDH: Lactate dehydrogenase
MAC: Membrane attack complex
PNH: Paroxysmal nocturnal hemoglobinuria
RBC: Red blood cell

## References

[1] Swann JW, Skelly BJ (2013). Systematic review of evidence relating to the treatment of immune-mediated hemolytic anemia in dogs. J Vet Intern Med.

[2] McCullough S (2003). Immune-mediated hemolytic anemia: understanding the nemesis. Vet Clin North Am Small Anim Pract.

[3] Johnson LR, Lappin MR, Baker DC (1999). Pulmonary thromboembolism in 29 dogs: 1985-1995. J Vet Intern Med.

[4] Scott-Moncrieff JC, Treadwell NG, McCullough SM, Brooks MB (2001). Hemostatic abnormalities in dogs with primary immune-mediated hemolytic anemia. J Am Anim Hosp Assoc.

[5] McManus PM, Craig LE (2001). Correlation between leukocytosis and necropsy findings in dogs with immune-mediated hemolytic anemia: 34 cases (1994-1999). J Am Vet Med Assoc.

[6] Reimer ME, Troy GC, Warnick LD (1999). Immune-mediated hemolytic anemia: 70 cases (1988-1996). J Am Anim Hosp Assoc.

[7] Grundy SA, Barton C (2001). Influence of drug treatment on survival of dogs with immune-mediated hemolytic anemia: 88 cases (1989-1999). J Am Vet Med Assoc.

[8] Carr AP, Panciera DL, Kidd L (2002). Prognostic factors for mortality and thromboembolism in canine immune-mediated hemolytic anemia: a retrospective study of 72 dogs. J Vet Intern Med.

[9] Weinkle TK, Center SA, Randolph JF, Warner KL, Barr SC, Erb HN (2005). Evaluation of prognostic factors, survival rates, and treatment protocols for immune-mediated hemolytic anemia in dogs: 151 cases (1993-2002). J Am Vet Med Assoc.

[10] Piek CJ, Junius G, Dekker A, Schrauwen E, Slappendel RJ, Teske E (2008). Idiopathic immune-mediated hemolytic anemia: treatment outcome and prognostic factors in 149 dogs. J Vet Intern Med.

[11] McAlees TJ (2010). Immune-mediated haemolytic anaemia in 110 dogs in Victoria, Australia. Aust 6 J.

[12] Goggs R, Dennis SG, Di Bella A, Humm KR, McLauchlan G, Mooney C (2015). Predicting outcome in dogs with primary immune-mediated hemolytic Anemia: results of a multicenter case registry. J Vet Intern Med.

[13] Brodsky RA (2015). Complement in hemolytic anemia. Blood.

[14] Brodsky RA (2014). Paroxysmal nocturnal hemoglobinuria. Blood.

[15] Hillmen P, Muus P, Duhrsen U, Risitano AM, Schubert J, Luzzatto L (2007). Effect of the complement inhibitor eculizumab on thromboembolism in patients with paroxysmal nocturnal hemoglobinuria. Blood.

[16] Hill A, Kelly RJ, Hillmen P (2013). Thrombosis in paroxysmal nocturnal hemoglobinuria. Blood.

[17] Kidd L, Mackman N (2013). Prothrombotic mechanisms and anticoagulant therapy in dogs with immune-mediated hemolytic anemia. J Vet Emerg Crit Care.

[18] Gigli I, Ruddy S, Austen KF (1968). The stoichiometric measurement of the serum inhibition of the first component of complement by the inhibition of immune hemolysis. J Immunol.

[19] Salvatierra A, Velasco F, Rodriguez M, Alvarez A, Lopez-Pedrera R, Ramirez R (1997). C1-esterase inhibitor prevents early pulmonary dysfunction after lung transplantation in the dog. Am J Respir Crit Care Med.

[20] Guerrero R, Velasco F, Rodriguez M, Lopez A, Rojas R, Alvarez MA (1993). Endotoxin-induced pulmonary dysfunction is prevented by C1-esterase inhibitor. J Clin Invest.

[21] Wouters D, Stephan F, Strengers P, de Haas M, Brouwer C, Hagenbeek A (2013). C1-esterase inhibitor concentrate rescues erythrocytes from complement-mediated destruction in autoimmune hemolytic anemia. Blood.

[22] Berentsen S, Sundic T (2015). Red blood cell destruction in autoimmune hemolytic anemia: role of complement and potential new targets for therapy. Biomed Res Int.

[23] Chan AW, Tetzlaff JM, Gotzsche PC, Altman DG, Mann H, Berlin JA (2013). SPIRIT 2013 explanation and elaboration: guidance for protocols of clinical trials. Bmj.

[24] Chan AW, Tetzlaff JM, Altman DG, Laupacis A, Gotzsche PC, Krleza-Jeric K (2013). SPIRIT 2013 statement: defining standard protocol items for clinical trials. Ann Intern Med.

[25] Shih AW, McFarlane A, Verhovsek M (2014). Haptoglobin testing in hemolysis: measurement and interpretation. Am J Hematol.

[26] Odhiambo CO, Otieno W, Adhiambo C, Odera MM, Stoute JA (2008). Increased deposition of C3b on red cells with low CR1 and CD55 in a malaria-endemic region of western Kenya: implications for the development of severe anemia. BMC Med.

[27] Hernandez DM, Goggs R, Behling-Kelly E (2018). In vitro inhibition of canine complement-mediated hemolysis. J Vet Intern Med.

