# Supplementary material for: C1 inhibitor in canine intravascular hemolysis (C1INCH): study protocol for a randomized controlled trial
Source: BMC Vet Res. 2019 Dec 30;15:475. doi: 10.1186/s12917-019-2220-2 (PMC6937664; doi:10.1186/s12917-019-2220-2)
Supplement: Supplementary file 1 — Additional file 1: C1INCH trial checklist (BMC Vet Res). A checklist for the C1INCH trial listing inclusion and exclusion criteria, sample requirements and the necessary steps for patient enrollment and randomization. [file 12917_2019_2220_MOESM1_ESM.pdf]

APPLY CASE STICKER HERE

C1-INCH Trial

- ☐ **Consent form signed**
- ☐ **Notify trial coordinators**
- ☐ **Notify Dr. Goggs**

INCLUSION CRITERIA

Primary intravascular IMHA

- ☐ Anemia (<30%) AND ≥1 of:
  - Positive in-saline agglutination test
  - Positive Coombs' test (if not agglutinating)
  - Moderate to marked spherocytosis
- ☐ Evidence of intravascular hemolysis
  Hemoglobinemia or hemoglobinuria

EXCLUSION CRITERIA

- ☐ <4kg OR >28kg
- ☐ 6 months old OR >10 years old
- ☐ Platelet count <30,000 cells / µL
- ☐ Underlying associated disease process
  e.g. Infectious disease, neoplasia, etc.
- ☐ Prior corticosteroids for ≥72h
- ☐ Prior immunosuppressives e.g. MMF, aza, cyclo

RECORD BASELINE (DAY 1) DATA

Age	_____	☐ MGCS	_____
Bodyweight	_____	☐ SpO_2	_____
Temperature	_____	☐ Fluid score	_____
Heart rate	_____	☐ Blood lactate	_____
Respiratory rate	_____	☐ Blood glucose	_____
Mentation score	_____	☐ SAP _____ / DAP _____ / MAP _____	

COLLECT BASELINE STUDY SAMPLES

☐ Serum:	2x red-top:	Separate serum, submit 1 for chemistry, split second into 2 and freeze at -20°C
☐ EDTA:	2x lilac top:	Submit 1 for CBC, submit 1 for erythrocyte bound C3b estimation`
☐ Citrate:	1x blue top:	Separate plasma, freeze at -20°C
☐ Heparin:	1x green top:	Separate plasma, freeze at -20°C

COMPLETE STANDARDIZED DIAGNOSTIC EVALUATION

- ☐ Complete blood count
- ☐ Serum chemistry
- ☐ Urinalysis
- ☐ Tick-borne disease serology or PCR testing
- ☐ In-saline agglutination OR Coombs' test
- ☐ 3-view thoracic radiographs
- ☐ Abdominal ultrasound

RANDOMIZE

- ☐ Login to site: https://www.sealedenvelope.com/simple-randomiser/v1/trials/c1inch
- ☐ Enter data, enter password, confirm inclusion/exclusions, enter location, then randomize

☐ Record randomization code here: ________________________________ If Pharmacy open, show code to Pharmacist ☐

☐ OOH: LVT to select corresponding package from Cubex, open, follow instructions. **DO NOT DISCLOSE TX TO DVM**

DAY 2

☐ Serum: 2x red-top: Separate serum, submit 1 for chemistry, split second into 2 and freeze at -20°C

☐ EDTA: 2x lilac top: Submit 1 for CBC, submit 1 for erythrocyte bound C3b estimation`

☐ Citrate: 1x blue top: Separate plasma, freeze at -20°C

☐ Heparin: 1x green top: Separate plasma, freeze at -20°C

DAY 3

☐ Serum: 2x red-top: Separate serum, submit 1 for chemistry, split second into 2 and freeze at -20°C

☐ EDTA: 2x lilac top: Submit 1 for CBC, submit 1 for erythrocyte bound C3b estimation`

☐ Citrate: 1x blue top: Separate plasma, freeze at -20°C

☐ Heparin: 1x green top: Separate plasma, freeze at -20°C

DAY 4

☐ Serum: 2x red-top: Separate serum, submit 1 for chemistry, split second into 2 and freeze at -20°C

☐ EDTA: 2x lilac top: Submit 1 for CBC, submit 1 for erythrocyte bound C3b estimation`

☐ Citrate: 1x blue top: Separate plasma, freeze at -20°C

☐ Heparin: 1x green top: Separate plasma, freeze at -20°C

DAY 5

☐ Serum: 2x red-top: Separate serum, submit 1 for chemistry, split second into 2 and freeze at -20°C

☐ EDTA: 2x lilac top: Submit 1 for CBC, submit 1 for erythrocyte bound C3b estimation`

☐ Citrate: 1x blue top: Separate plasma, freeze at -20°C

☐ Heparin: 1x green top: Separate plasma, freeze at -20°C

FOLLOW-UP - DAY 7

☐ IF DISCHARGED... Call client

☐ Determine and record patient status (Alive, Died, Euthanized) ________________________________

☐ Adverse events (Y/N) ________________________________. Detail separately if yes.

FOLLOW-UP - DAY 14

☐ IF DISCHARGED... Call client

☐ Determine and record patient status (Alive, Died, Euthanized) ________________________________

☐ Adverse events (Y/N) ________________________________. Detail separately if yes.

FOLLOW-UP - DAY 28

☐ IF DISCHARGED... Call client

☐ Determine and record patient status (Alive, Died, Euthanized) ________________________________

☐ Adverse events (Y/N) ________________________________. Detail separately if yes.
