# Supplementary material for: C1 inhibitor in canine intravascular hemolysis (C1INCH): study protocol for a randomized controlled trial
Source: BMC Vet Res. 2019 Dec 30;15:475. doi: 10.1186/s12917-019-2220-2 (PMC6937664; doi:10.1186/s12917-019-2220-2)
Supplement: Supplementary file 2 — Additional file 2: C1INCH trial forms. Data collection sheet for the C1INCH trial including instruments for collection of follow-up metrics. [file 12917_2019_2220_MOESM2_ESM.pdf]

C1 Inhibitor in Canine Intravascular Hemolysis (C1INCH)

Date: ____ Time: ______________________________________

Patient: ____ Client: ___________________________________

Hospital Case Number: ___________________________ Phone: ____________________________________

Study Site: ___________________________________ Consent signed: YES ☐

Breed: ____ Sex/Neuter status: _______________________

Age/DOB: _____________________________________ Bodyweight: ____________________________

Prior medical history: ____

____

____

Day 1 checklist:

☐ Study blood samples collected
☐ CBC
☐ Thoracic radiography
☐ Urinalysis
☐ In-saline agglutination test or Coombs' test
☐ Serum chemistry
☐ Abdominal ultrasound
☐ Tick-borne disease testing

Randomization code: _______________________ ☐ Randomized in-hours ☐ Randomized out-of-hours

Day 2 checklist:

☐ Study blood samples collected

Day 3 checklist:

☐ Study blood samples collected

Day 4 checklist:

☐ Study blood samples collected

Day 5 checklist:

☐ Study blood samples collected

Record discharge status:

☐ Survived to hospital discharge ☐ Died ☐ Euthanized (Reason: _________________________________)

Date of discharge: ___________________________ Duration of hospitalization: ___________________________

RBC transfusions: _______________________ Volume of RBCs (mL/kg) _______________________________

☐ Drug administered? Adverse events at time of administration? ☐ Y ☐ N

If yes, provide details: ____

____

☐ Rescue therapy administered? Drug(s) administered: ______________________________

Provide details regarding rationale: ____

____

C1 Inhibitor in Canine Intravascular Hemolysis (C1INCH)

Follow-up +7d

What is the dog's clinical status? ____

What medications is the dog taking at home? ____
____

Current clinical signs? ____
____

Follow-up +14d

What is the dog's clinical status? ____

What medications is the dog taking at home? ____
____

Current clinical signs? ____
____

Follow-up +28d

What is the dog's clinical status? ____

What medications is the dog taking at home? ____
____

Current clinical signs? ____
____

Any other notes:
